# Overview of changes in the Clinical and Laboratory Standards Institute Performance Standards for Antimicrobial Susceptibility Testing: M100 32nd and 33rd editions

**DOI:** 10.1128/jcm.01623-23

**Published:** 2025-08-07

**Authors:** Audrey N. Schuetz, Andrea Ferrell, Janet A. Hindler, Romney Humphries, April M. Bobenchik

**Affiliations:** 1 Department of Laboratory Medicine and Pathology, Mayo Clinic College of Medicine and Science 12270 , Rochester, Minnesota, USA; 2 R&D, BD Life Sciences 157841 , Sparks, Maryland, USA; 3 Public Health Laboratories, Los Angeles County Department of Public Health, Los Angeles, California, USA; 4 Department of Pathology, Microbiology and Immunology, Vanderbilt University Medical Center 12328, Nashville, Tennessee, USA; 5 Department of Pathology and Laboratory Medicine, Penn State Hershey Medical Center, Hershey, Pennsylvania, USA; 6 Deparment of Pathology, Division of Clinical Pathology, Penn State College of Medicine, Hershey, Pennsylvania, USA; Boston Children's Hospital, Boston, Massachusetts, USA

**Keywords:** CLSI, M100, antibiotic, antimicrobial agents, susceptibility testing, minimal inhibitory concentration, disk diffusion, Kirby-Bauer, breakpoints, quality control

## Abstract

The Clinical and Laboratory Standards Institute Subcommittee on Antimicrobial Susceptibility Testing (AST) publishes annual updates to the M100 *Performance Standards for Antimicrobial Susceptibility Testing*. This important document contains key information critical to the laboratory’s performance of accurate and current AST. This minireview will highlight and provide supporting data for major changes in the M100 32nd and 33rd editions published in February 2022 and March 2023, respectively. New and revised breakpoints for gram-negative and gram-positive organisms are explained. Proper use of the restructured M100 Table 1 guidance for antimicrobial agents to be considered for testing and reporting is outlined. Reporting guidance for multidrug-resistant gram-negative bacilli is discussed. Other topics within this minireview include additional agents to test and report for direct blood disk diffusion of gram-negative bacilli and quality control range changes and troubleshooting. The minireview concludes with other issues under consideration by the AST Subcommittee.

## INTRODUCTION

The Clinical and Laboratory Standards Institute (CLSI) is a volunteer-led body composed of representatives from the healthcare professions, government, and industry, which publishes standards and guidelines for laboratories. CLSI’s Subcommittee on Antimicrobial Susceptibility Testing (AST) describes methods, quality control (QC), breakpoints, and interpretive categories for AST reporting. In addition, the AST Subcommittee creates educational activities that are coordinated by the Outreach Working Group (ORWG); this minireview is one such activity. The M100 *Performance Standards for Antimicrobial Susceptibility Testing* document, developed by the AST Subcommittee, provides guidance for laboratories on breakpoints, quality control, test conditions and methods, and a wide range of other useful information such as intrinsic resistance, body site reporting restrictions, and handling of genotypic versus phenotypic resistance discrepancies, among others. Updates to the M100 document occur annually. This minireview will highlight major changes to the M100 32nd and 33rd editions, published in February 2022 and March 2023, respectively (1, 2).

## BREAKPOINTS

Breakpoints discussed below are revisions of prior breakpoints, with the exception of plazomicin, for which new breakpoints have been set. New and revised breakpoints are presented in Table 1.

**TABLE 1:** New/updated breakpoints in CLSI M100 32nd or 33rd edition*^a^*^,^*^b^*^,^*^e^*^,*g*^

Organism/organism group and antimicrobial agent	Disk content (µg)	Interpretive categories and zone diameter breakpoints (mm)			Interpretive categories and MIC breakpoints (µg/mL)			FDA breakpoints same as CLSI breakpoints?^*c*^
		S	I	R	S	I	R	
Enterobacterales								
Amikacin^*b*^	30	≥20	17–19^*f*^	≤16	≤4	8^*f*^	≥16	No
Cefiderocol*^a^^,^^d^*	30	≥16	9–15^*f*^	≤8	≤4	8^*f*^	≥16	Yes
Ceftolozane-tazobactam*^a^*^,*d*^	30/10	≥22	19–21^*f*^	≤18	≤2/4	4/4^*f*^	≥8/4	Yes
Gentamicin^*b*^	10	≥18	15–17^*f*^	≤14	≤2	4^*f*^	≥8	No
Piperacillin-tazobactam^*b*^	100/10	≥25	21–24 (SDD)	≤20	≤8/4	16/4 (SDD)	≥32/4	Partial: I not SDDs
Plazomicin^*b*^	30	≥18	15–17^*f*^	≤14	≤2	4^*f*^	≥8	Yes
Tobramycin^*b*^	10	≥17	13–16^*f*^	≤12	≤2	4^*f*^	≥8	No
*Pseudomonas aeruginosa*								
Amikacin^*b*^	30	≥17	15–16^*f*^	≤14	≤16	32^*f*^	≥64	No
Gentamicin^*b*^	10	–	–	–	–	–	–	No
Piperacillin-tazobactam^*b*^	100/10	≥22	18–21^*f*^	≤17	≤16/4	32/4^*f*^	≥64/4	Yes
Tobramycin^*b*^	10	≥19	13–18^*f*^	≤12	≤1	2^*f*^	≥4	No
*Acinetobacter* spp.								
Cefiderocol*^a^*^,*d*^	30	≥15	–	–	≤4	8	≥16	No
*Stenotrophomonas maltophilia*								
Cefiderocol^*a*^	30	≥15	–	–	≤1	–	–	Partial: recognizes MIC but not disk diffusion breakpoints
*Haemophilus influenzae*								
Amoxicillin-clavulanate^*a*^	20/10	–	–	–	≤2/1	4/2	≥8/4	Yes
Lefamulin^*a*^^,^^*d*^	20	≥18	–	–	≤2	–	–	Yes
*Streptococcus pneumoniae*								
Lefamulin*^a^*^,*d*^	20	≥19	–	–	≤0.5	–	–	Yes

^*a*^ Change occurred in M100 32nd edition.

^*b*^ Change occurred in M100 33rd edition.

^*c*^ https://www.fda.gov/drugs/development-resources/fda-recognized-antimicrobial-susceptibility-test-interpretive-criteria (accessed 18 June 2025).

^*d*^ MIC breakpoints were previously established and not new in M100 32nd edition or M100 33rd edition.

^*e*^ DD, disk diffusion; MIC, minimal inhibitory concentration; I, intermediate; R, resistant; S, susceptible; and SDD, susceptible dose dependent.

^*f*^ Concentrates in urine.

^*g*^ "-" indicates lack of a breakpoint at the interpretive category.

### Aminoglycosides, including plazomicin, for Enterobacterales and *Pseudomonas aeruginosa*

In 2022, CLSI underwent a major review of the aminoglycoside breakpoints for Enterobacterales and *P. aeruginosa* to update gentamicin, tobramycin, and amikacin breakpoints and publish new breakpoints for plazomicin for Enterobacterales. The aminoglycoside class binds the 30S ribosome of many bacteria, inhibiting protein synthesis. Activity is oxygen dependent, resulting in no activity against anaerobic bacteria. Other notable organisms for which aminoglycosides show gaps in activity include *Salmonella*, *Shigella*, *Stenotrophomonas*, *Burkholderia*, *Streptococcus,* and *Enterococcus*. Resistance to the aminoglycosides in gram-negative bacteria occurs by three primary pathways: enzymatic inactivation of the aminoglycoside by the bacterium through acetylation, phosphorylation, or adenylation; alteration of the bacterial ribosomal target site through methylation; and decrease in cell wall permeability to the aminoglycosides, particularly for *P. aeruginosa*.

Breakpoints for the aminoglycosides were first published by CLSI (then called the National Committee for Clinical Laboratory Standards) in 1979. At the time, very little data were available on the pharmacodynamics (PD) of the aminoglycosides, and the breakpoints were based on wild-type minimal inhibitory concentration (MIC) distributions and minimal pharmacokinetic (PK) information. Concerns were noted about the breakpoints as early as the 1980s, as achievable serum levels of aminoglycosides, when administered using package insert dosing regimens, were viewed as too low to cover all susceptible isolates. However, no changes to the breakpoints were made. In 2018, CLSI evaluated plazomicin, a novel aminoglycoside, which led to the recognition that breakpoints for the other aminoglycosides likely required revision. Specifically, contemporary aminoglycoside dosing, using high-dose, once-daily extended-interval regimens as opposed to multiple daily dosing as listed in the prescribing information, has been shown to mitigate adverse events associated with aminoglycoside use (3–5). It also results in a different exposure of aminoglycoside, implying that a breakpoint evaluation was needed, per CLSI M23 criteria (6).

The focus of CLSI revisions was on gentamicin, tobramycin, and amikacin for both Enterobacterales and *P. aeruginosa*. Several additional aminoglycosides, such as kanamycin, netilmicin, and streptomycin, with breakpoints published in M100, are available in the United States but are rarely used today due to very little activity against Enterobacterales and *P. aeruginosa*. As such, these antimicrobials were not considered in CLSI’s recent revisions to the aminoglycoside class. In addition, breakpoints for *Acinetobacter* spp., the non-Enterobacterales group, and *Staphylococcus* spp. were not evaluated due to limited data for the former two organism groups and the use of aminoglycosides as part of combination therapy for the latter.

During the breakpoint setting discussions, epidemiological cut-off values (ECVs) for each organism group and aminoglycoside were determined and are listed in Table 2. These ECVs define the high-end MIC for the wild-type population, i.e., MICs at or below which resistance mechanisms are not detectable. Across the Enterobacterales, however, there are differences in ECVs by species. Some species demonstrate elevated ECVs for select aminoglycosides. In particular, *Serratia marcescens* has an ECV of 8 µg/mL to tobramycin, which is above the ECV and the susceptible breakpoint of ≤2 µg/mL for the Enterobacterales and thus may often test resistant to tobramycin. Nonetheless, tobramycin should be reported as it tests for *S. marcescens*. Laboratories should adhere to guidance on aminoglycoside reporting for the various members of Enterobacterales provided in the intrinsic resistance tables in Appendix B of M100.

**TABLE 2:** Enterobacterales and *Pseudomonas aeruginosa* minimal inhibitory concentration breakpoints for gentamicin, tobramycin, and amikacin*^a^*^,^*^d^*^,*e*^

Organism	MIC (µg/mL)			
	ECV	Updated clinical breakpoints		
		S	I	R
Enterobacterales				
Gentamicin	2	≤2	4	≥8
Tobramycin	2	≤2	4	≥8
Amikacin	8	≤4	8	≥16
*P. aeruginosa*				
Gentamicin^*b*^	8	–	–	–
Tobramycin	2	≤1	2	≥4
Amikacin^*c*^	16	≤16	32	≥64

^*a*^ Updated in M100 33rd edition; disk diffusion breakpoints were also updated (see Table 1).

^*b*^ Although *P. aeruginosa* does not qualify as intrinsically resistant to gentamicin based on the CLSI definition, a breakpoint does not exist, and it should not be considered a viable treatment option for infections due to *P. aeruginosa* at this time.

^*c*^ Amikacin is recommended for use only in uncomplicated urinary tract infections.

^*d*^ S, susceptible; I, intermediate; and R, resistant.

^*e*^ "-" indicates lack of a breakpoint at the interpretive category.

Extensive pharmacokinetic-pharmacodynamic (PK-PD) data were evaluated for Enterobacterales and *P. aeruginosa*. The ratio of area under the concentration-time curve to MIC (AUC:MIC) was deemed most predictive of aminoglycoside activity, in contrast to more traditional aminoglycoside evaluations, where the maximum concentration (*C*_max_) to MIC ratio was used based on data from murine neutropenic thigh models (7). The data for this work were compiled by colleagues at the United States Committee on Antimicrobial Susceptibility Testing (USCAST) (8). In essence, the USCAST document demonstrated that achieving a 2-log colony-forming unit (CFU) reduction from baseline in a murine thigh or lung model required exposures far higher than what is achievable with safe levels of prescribed gentamicin, tobramycin, and amikacin for isolates of Enterobacterales and *P. aeruginosa* with MICs at or below the ECV. A 1-log CFU reduction was similarly unachievable, but net bacterial stasis endpoints could be reasonably achieved. Thus, the revised CLSI breakpoints are most appropriate for infections where stasis endpoints are sufficient: those with low bacterial burden and good source control, low host comorbidities, and for which an aminoglycoside is used as part of combination therapy (8).

While the use of a stasis endpoint predicted a susceptible breakpoint below the ECV for the Enterobacterales, data evaluated by CLSI indicated that achieving PK-PD stasis endpoints was more challenging for *P. aeruginosa*. In particular, no susceptible breakpoint could be defined for gentamicin against *P. aeruginosa*. A maximum gentamicin MIC of 1.0 µg/mL for *P. aeruginosa* was predicted to achieve the AUC:MIC exposure required for bacterial stasis, but this MIC is far below the *P. aeruginosa* ECV of 8 µg/mL. In other words, the data demonstrated that wild-type isolates (with MICs ≤8 µg/mL) were not treatable with gentamicin. Since increasing the dose of gentamicin is not possible due to the risk for toxicity, CLSI removed gentamicin breakpoints for *P. aeruginosa*, which aligns with the removal of gentamicin breakpoints for *P. aeruginosa* by the European Committee on Antimicrobial Susceptibility Testing (EUCAST) in recent years. According to CLSI, *P. aeruginosa* is not considered intrinsically resistant to gentamicin; however, given the lack of a breakpoint, it is not considered a viable treatment option for infections due to *P. aeruginosa* at this time.

PK-PD stasis endpoints were similarly difficult to achieve for amikacin against *P. aeruginosa*. Bacterial stasis for amikacin in the serum was predicted to occur up to a maximum MIC of 4.0 µg/mL. However, the ECV is 16 µg/mL, which is two doubling dilutions above this value. In this case, consideration was given to the ability of amikacin to concentrate in the urine and renal cortex, leading to the possibility of treating infections originating in the urinary tract with amikacin. As such, CLSI has updated the amikacin breakpoint for isolates recovered from urine only; this breakpoint was designated as “U” in the M100 33rd edition.

Finally, for tobramycin, an 82.1% probability of achieving bacterial stasis was predicted at an MIC of 1 µg/mL for *P. aeruginosa*. While the ECV for this antimicrobial is 2 µg/mL, the tobramycin MICs of only a small fraction of isolates (5%–10%) are predicted to measure 2 µg/mL. Since the clinical ramifications of deleting all aminoglycosides for the treatment of *P. aeruginosa* would be significant, the committee’s consensus decision was to set the tobramycin breakpoint one doubling dilution below the ECV despite the disadvantages of cutting into the wild-type population; this was deemed to be an acceptable compromise.

Aminoglycoside *P. aeruginosa* breakpoints differ among breakpoint setting organizations. The United States Food and Drug Administration (FDA), CLSI, and EUCAST each have different *P. aeruginosa* breakpoints for amikacin and tobramycin. Gentamicin *P. aeruginosa* breakpoints still exist for FDA and USCAST, while they do not for CLSI and EUCAST. The various breakpoints, or the lack thereof, are indicative of difficulties in setting breakpoints for aminoglycosides. Laboratories should discuss these issues with their clinical providers, infectious diseases pharmacists, and other healthcare providers, review applicable treatment guidelines released by professional organizations, and together determine approaches to testing and reporting of aminoglycosides for *P. aeruginosa*.

Plazomicin received FDA approval in 2018 for the treatment of complicated urinary tract infections in adults. It was developed to treat infections caused by extended-spectrum beta-lactamase (ESBL)-producing or carbapenem-resistant Enterobacterales. Its structure protects it from degradation by most aminoglycoside-modifying enzymes. Plazomicin has little added activity compared to other aminoglycosides against *P. aeruginosa* and *Acinetobacter* spp., and thus, there are no breakpoints for those organisms. MIC and disk diffusion breakpoints for plazomicin and Enterobacterales (excluding the Morganellaceae family, which includes *Proteus*, *Providencia,* and *Morganella* spp.) were published in the M100 33rd edition. Isolates of the Morganellaceae family display elevated MICs against plazomicin, which extend beyond the wild type.

Updated aminoglycoside breakpoints are listed in Table 2. Corresponding disk diffusion breakpoints were established, which showed excellent correlation with MIC results. Laboratories should discuss these changes with their institution’s antimicrobial stewardship programs (ASPs), as well as with infectious diseases clinicians and pharmacy. The FDA has not yet recognized these CLSI revised aminoglycoside breakpoints; as a result, commercial manufacturers cannot obtain FDA clearance for the current CLSI Enterobacterales and *P. aeruginosa* aminoglycoside breakpoints. Laboratories may consider off-label adoption of the breakpoints following a validation study, if their test system includes MIC concentrations low enough to accommodate the breakpoints (Table 3). Interim steps until the FDA recognizes CLSI breakpoints may include suppressing gentamicin results from *P. aeruginosa* or categorizing gentamicin as “R” regardless of the MIC or disk diffusion zone measurement. Laboratories should also consider reporting amikacin only for urinary isolates of *P. aeruginosa*. As mentioned, disk diffusion test performance with the revised breakpoints was found to be excellent, and laboratories unable to adopt the breakpoints on their automated systems may consider performing disk diffusion in select cases, as discussed with ASP. A Centers for Disease Control and Prevention (CDC) and FDA Antimicrobial Resistance (AR) Bank isolate panel of 30 organisms is available to aid laboratories in validating testing for plazomicin.

**TABLE 3:** Availability of aminoglycoside dilutions on automated antimicrobial susceptibility testing systems to accommodate updated CLSI aminoglycoside breakpoints

Organism/antimicrobial agent	BD Phoenix	Beckman Coulter MicroScan	bioMérieux Vitek2
Enterobacterales			
Amikacin	No, lowest dilution is 8 µg/mL	Yes, on certain panels	Yes
Gentamicin	Yes	Yes	Yes
Tobramycin	Yes	Yes	Yes
*Pseudomonas aeruginosa*			
Amikacin	Yes	Yes	Yes
Tobramycin	No, lowest dilution is 2 µg/mL	Yes, on certain panels	Yes

### Piperacillin-tazobactam for Enterobacterales and *P. aeruginosa*

CLSI revised the piperacillin-tazobactam (TZP) clinical breakpoints for the Enterobacterales in 2022 (9) and those of *P. aeruginosa* in 2023 (10). These revisions were informed by an in-depth review of clinical data and the extensive body of PK-PD literature that has been published for TZP over the past four decades. Rationale documents are available for these changes from CLSI (www.clsi.org) and have been published in *Clinical Infectious Diseases* (9, 10).

TZP was first approved for human use in the United States in 1993, at which time breakpoints for Enterobacteriaceae, *P. aeruginosa,* and *Acinetobacter* spp. were the same. CLSI revised *P. aeruginosa* breakpoints in 2012, when the susceptible breakpoint was updated from ≤64 to ≤16 µg/mL, but the Enterobacterales breakpoint had not undergone review since 1993. The major incentive for a review was the publication of the MERINO trial, which failed to achieve a non-inferiority endpoint for TZP compared to meropenem for the treatment of ceftriaxone not susceptible (e.g., intermediate and resistant) *Escherichia coli* and *Klebsiella* spp. (11). *Post hoc* analysis of this study identified that TZP was erroneously reported as susceptible by the commercial AST methods used in the trial, and reference broth microdilution (BMD) MIC results showed an association of MIC >16 µg/mL and mortality in the trial (12). PD data indicated that a time above MIC of 50% is required for bactericidal activity of TZP. For patients treated with TZP using routine dosing (i.e., 3.375–4.5 g q6h as a 30-minute infusion), PK-PD analyses suggest that MICs up to 8 µg/mL can achieve this endpoint. However, if an extended infusion strategy is used (i.e., 4.5 g q6h as a 3-h infusion or q8h as a 4-h infusion), MICs up to 16 µg/mL are predicted to achieve the target. Based on these data, the TZP Enterobacterales breakpoints were revised to ≤8/4 µg/mL (susceptible), 16/4 µg/mL (susceptible-dose dependent using an extended infusion strategy), and ≥32/4 µg/mL (resistant).

Several features differentiate the activity of TZP against *P. aeruginosa* compared to the Enterobacterales. First, unlike the Enterobacterales, the tazobactam component does not enhance piperacillin activity in *P. aeruginosa*, as class A beta-lactamases inhibited by tazobactam for Enterobacterales are rarely expressed in *P. aeruginosa* (13, 14). However, *P. aeruginosa* has a higher wild-type piperacillin MIC compared to the majority of Enterobacterales: the *P. aeruginosa* ECV is 16 µg/mL, while the Enterobacterales ECV is 8 µg/mL. The PK-PD target for piperacillin against *P. aeruginosa* is also 50% time above the MIC, and PK-PD endpoints are the same as those described above for the Enterobacterales (i.e., MIC of 8 µg/mL is achievable using 30-minute infusions, whereas MIC of 16 µg/mL requires extended infusion strategies) (10). As such, a susceptible breakpoint of ≤8/4 µg/mL was considered by CLSI but ultimately rejected as this would bisect the wild-type population. Rather, the susceptible breakpoint of ≤16/4 µg/mL was left untouched, but the intermediate category was updated from 32/4-64/4 µg/mL to 32/4 µg/mL, as no data suggested that TZP MICs >16/4 µg/mL could be treated, regardless of the dosing regimen used (Table 1). The revised *P. aeruginosa* TZP intermediate category (32/4 µg/mL) accounts for technical variability and the ability of the agent to concentrate in the urine (as indicated by an I^ in M100).

With the changes to the TZP breakpoints, it is worth reviewing the multiple meanings for the intermediate category. According to both CLSI and FDA, “I” accounts for an MIC that may predict treatment success, if the infection is located in a body site where the antimicrobial agent physiologically concentrates (e.g., the urine) and is specifically denoted in M100 with an I^, or if higher exposure to the antimicrobial via increased concentrations or alternative dosing regimens can be achieved. The distinction between “I” and susceptible dose dependent (SDD) is nuanced; SDD refers to increasing the exposure through dosing, whereas “I” includes both alternative dosing regimens and physiological concentration of the antimicrobial in select compartments of the human body, achievable with routine dosing. However, “I” also serves as a buffer zone to account for technical variability in susceptibility tests and may also indicate if the activity of the antimicrobial agent is uncertain (i.e., in the case of colistin).

The US FDA has recognized the updated TZP breakpoints for Enterobacterales and *P. aeruginosa,* except that the FDA categorizes an MIC of 16/4 µg/mL for Enterobacterales as “I” instead of SDD. Laboratorians must be aware, however, that unacceptable error rates (e.g., higher-than-desired very major errors or lower-than-desired categorical agreement) have been noted with different commercial AST systems when applying the updated CLSI Enterobacterales piperacillin-tazobactam breakpoints (15). An alternate testing method may be necessary for certain antimicrobial agent/organism combinations if the error rates are too high. A CDC and FDA AR Bank isolate set is available to aid with validation. It is also worth noting that CLSI has identified suboptimal performance of the TZP disk when applying the revised CLSI breakpoints for the Enterobacterales (16), which suggests a reformulation of the disk mass may be needed. Studies jointly undertaken by CLSI and EUCAST to reassess the correct disk mass for piperacillin-tazobactam are currently underway.

### Amoxicillin-clavulanate for *Haemophilus influenzae* and *Haemophilus parainfluenzae*

There has been a several-year effort by CLSI to review the aminopenicillin breakpoints since these antimicrobial agents are used to cover many clinical indications with many different dosing regimens. The breakpoints for amoxicillin-clavulanate were originally set in the 1980s using the sparse data available at the time, so CLSI reviewed the breakpoints for amoxicillin-clavulanate and the Enterobacterales, *Enterococcus* spp., *Haemophilus* spp., *Streptococcus pneumoniae*, *Streptococcus* spp. β-hemolytic group, *Streptococcus* spp. viridans group, and *Neisseria meningitidis*. The retrospective review, which primarily included PK-PD and available outcome data, supported nearly all the currently published breakpoints for amoxicillin-clavulanate (as well as for ampicillin, ampicillin-sulbactam, and amoxicillin), and dosing comments were placed throughout the M100 Table 2 to explain the dosage regimens on which the breakpoints were established. There was one breakpoint that was found to be in need of an update: the MIC susceptible breakpoint of 4/2 µg/mL for amoxicillin-clavulanate and *H. influenzae* and *H. parainfluenzae* was not achievable with oral dosing by PK-PD, which was problematic as the only available form of amoxicillin-clavulanate in the United States is the oral formulation. Therefore, the MIC breakpoints for amoxicillin-clavulanate for *H. influenzae* and *H. parainfluenzae* were changed in M100 32nd edition from S ≤ 4/2 µg/mL; R ≥ 8/4 µg/mL to S ≤ 2/1 µg/mL; I = 4/2 µg/mL; and R ≥ 8/4 µg/mL. The zone diameter breakpoints were removed until review of additional disk correlate data with the current dosage regimen occurs.

### Cefiderocol

Cefiderocol is a novel cephalosporin conjugated to an iron-chelating catechol moiety. It is known as the “Trojan horse” drug, as it acts as a siderophore by binding to free iron within the environment and using the bacterial active ion-specific transport system to enter the cell. Upon entering the periplasmic space, the cephalosporin portion of cefiderocol is released. Cefiderocol is active against ESBLs and carbapenemases, including metallo-beta-lactamases. Its mode of functionality requires that MIC testing of the agent be performed using iron-depleted cation-adjusted Mueller-Hinton broth. Investigational MIC and disk diffusion breakpoints were first published in M100 in 2019 and 2020, respectively. CLSI reevaluated these investigational breakpoints in 2021 using new clinical trial data from Shionogi, leading to the revision of the disk diffusion breakpoints for the Enterobacterales, *Acinetobacter* spp., and *Stenotrophomonas maltophilia* in the M100 32nd edition. The MIC breakpoints for *S. maltophilia* were also revised, and the agent was placed in Group B for testing of the indicated organisms in Table 1A in M100 32nd edition, but later moved to Tier 3 in Table 1 of the M100 33rd edition. At the time of this article’s publication, the FDA recognizes the current CLSI breakpoints for cefiderocol and the Enterobacterales but maintains different breakpoints for *P. aeruginosa* and *A. baumannii* complex. The FDA recognizes the MIC breakpoints for *S. maltophilia* but has different disk diffusion breakpoints.

There are several noteworthy issues with cefiderocol testing, including difficult-to-interpret MIC and disk diffusion endpoints (trailing in BMD and inner colonies in disk diffusion), small differences in test inoculum resulting in major differences in MICs, and poor reproducibility of BMD and disk diffusion results for *A. baumannii* isolates with MICs > 2 µg/mL (17, 18). For testing of cefiderocol by disk diffusion for *A. baumannii*, zone diameter results <15 mm are uninterpretable and should not be reported. Thus, if zone diameters <15 mm are obtained for *Acinetobacter*, MIC testing should be performed. As a result of testing issues, comments were placed throughout Table 2 in M100 that warn that the accuracy and reproducibility of cefiderocol testing are markedly affected by iron concentration and inoculum preparation and may vary by disk and media manufacturer. Both false-resistant and false-susceptible results may occur. There are some commercially available test methods (both based on broth microdilution and disk diffusion) for cefiderocol. Appendix I in M100 33rd edition (now Appendix H in M100 35th edition) also has detailed instructions for the preparation of broth microdilution plates using the reference method.

### Lefamulin

Lefamulin is the first systemic antibiotic of the pleuromutilin class. It received FDA approval in 2019 for the treatment of adults with community-acquired bacterial pneumonia (CABP). CLSI breakpoints for lefamulin were first published in 2021. However, when the disk diffusion breakpoints were originally set by CLSI, there were very few isolates within the nonsusceptible category. Additional data obtained when testing an expanded challenge set of organisms against the reference BMD method were subsequently gathered from several different studies. As a result, the susceptible disk diffusion breakpoint for *H. influenzae* was altered by 1 mm, from ≥17 to ≥18 mm. The susceptible breakpoint for *S. pneumoniae* increased by 2 mm to ≥19 mm. These updated breakpoints resulted in fewer very major errors. Clinical use of lefamulin is most often empiric, based on high susceptibility rates of CABP organisms and the limited value of sputum culture in the diagnosis of CABP (19). FDA-cleared disks are available from Hardy Diagnostics and Becton Dickinson (BD). FDA-cleared MIC methods include Sensititre BMD panels and MIC test strips (MTS, Liofilchem) for agar gradient diffusion.

## METHODS AND TESTING CONSIDERATIONS

Testing conditions, such as medium, inoculum, and incubation conditions, are provided in Table 2 of the M100 document, while updated QC organisms and/or ranges are provided in Tables 4 and 5 of M100 (2). It is worth noting that laboratories that use an FDA-cleared or -approved AST device, such as agar gradient diffusion, must follow the manufacturer’s instructions for testing conditions.

### Reformatting of M100 Tables 1 “Antimicrobial Agents that Should be Considered for Testing and Reporting by Microbiology Laboratories”

Tables 1 have been an integral part of the M100 since 1972. These tables include agents that show clinical efficacy, achieved consensus agreement on first-line choices, and have FDA clinical indications for use. M100 Tables 1 are to be used as resources for clinical laboratories to help guide appropriate testing and reporting of antimicrobial agents and to be shared with relevant ASP stakeholders. Tables 1 were significantly revised in the M100 33rd edition to reflect the addition of newer antimicrobial agents, the emergence of novel mechanisms of resistance, and the increasing prevalence of multidrug-resistant organisms. The revised tables also place a stronger emphasis on antimicrobial stewardship practices. Other publications have provided a detailed review of M100 Table 1 revisions with suggestions for incorporation into routine clinical laboratory practice (20).

In brief, changes to the instructions for use include the expansion of group/tiered definitions and clarifications to the selective and cascade reporting definitions. Antimicrobial agents within M100 Tables 1 were originally classified into three groups: A, B, and C. However, agents are now grouped into four tiers: 1, 2, 3, and 4. Group or tier assignments were based on the activity of an agent (e.g., narrow or broad spectrum), its intended use, and a suggestion for reporting the agent routinely or following specific rules. The expansion from three to four groups was required to account for the number of newer antimicrobial agents and the increasing prevalence of antimicrobial resistance. The tiered definitions are similar to the old Group A, B, and C definitions. Tier 1 agents are appropriate for routine, primary testing and reporting. Tier 2 agents are appropriate for routine, primary testing but may be reported following cascading rules established by each individual institution. Tier 3 is a new tier to account for institutions that may have high levels of multidrug-resistant organisms; these agents are appropriate for routine, primary testing and reporting and should follow institution-specific cascade rules. Tier 4 agents are generally reserved for agents that are tested and reported on request due to the availability of a preferred agent or a patient’s underlying condition, including allergies. In addition, M100 Tables 1 also include notations of agents to be tested routinely only on isolates from urinary sources and reported as appropriate. Some agents with clinical breakpoints included in M100 Tables 2 are not in Tables 1, as they are not considered candidates for routine testing and reporting because they do not have FDA clinical indications for use in the United States. These agents are designated with an asterisk in Tables 2 or noted with an “Inv” (investigational) if they have not yet been approved by the FDA.

Selective reporting refers to the reporting of results for specific antimicrobial agents based on defined criteria that are unrelated to the overall susceptibility profile of the organism. In other words, results are reported based on organism identification, body site source, clinical setting, or patient demographics. An example of selective reporting is withholding aminoglycoside results on *Salmonella* spp. or *Shigella* spp. due to their inability to achieve clinical efficacy *in vivo*. Unlike selective reporting, cascade reporting is based on the results of the overall antimicrobial susceptibility profile of an isolate. When applying cascade reporting, results for secondary or broader spectrum agents are only reported if the isolate is resistant to primary or narrow-spectrum agents in the same or similar antimicrobial class. Cascade reporting can be either within the same tier (e.g., for Tier 2 Table 1A Enterobacterales: if cefepime tests resistant, then the carbapenems would be reported) or between tiers (e.g., for Tier 2 to Tier 3 Table 1A Enterobacterales: if both cefepime and the carbapenems test resistant, then a newer beta-lactam combination agent would be reported). The layout of the tables has changed from a vertical format to a horizontal format. Antimicrobial agents are arranged left to right corresponding to their tiered definitions 1 through 4. The horizontal presentation helps facilitate cascade reporting across the tiers from a narrow-spectrum agent in Tier 1 to a broad-spectrum agent in Tier 3.

### Testing and reporting recommendations for multidrug-resistant gram-negative bacilli

The M100 contains comments throughout the tables, which are mainly intended for clinical laboratorians. These comments highlight potential AST problems and/or add clarification to specific testing issues. The M100 32nd edition included a revision to the beta-lactam combination agent comment for Enterobacterales, *P. aeruginosa*, *Acinetobacte*r spp., Non-Enterobacterales, *H. influenzae*, *H. parainfluenzae*, and anaerobes. The new comment states, “Organisms that test susceptible to the beta-lactam agent alone are also considered susceptible to the beta-lactam combination agent. However, organisms that test susceptible to the beta-lactam combination agent cannot be assumed to be susceptible to the beta-lactam agent alone.” This comment replaced the previous imipenem-relebactam surrogate testing comment that was specific to this agent. The intention of this new comment is to emphasize that predicting susceptibility to the beta-lactam combination agent from the susceptible beta-lactam agent alone applies to all beta-lactam combination agents—not just imipenem-relebactam.

In 2010, CLSI lowered the Enterobacterales cephalosporin and carbapenem breakpoints, eliminating the requirement to perform specific resistance mechanism identification for ESBLs or carbapenemase producers for laboratories that adopted the lower breakpoints. At the time, the lower breakpoints were considered sufficient to accurately detect wild-type organisms lacking acquired resistance mechanisms. However, with the publication of the MERINO trial data that focused on infections caused by ESBL-producing isolates of Enterobacterales and the development of new antimicrobials that target specific mechanisms of resistance, the need to identify the mechanism of certain types of resistance has resurfaced (11). The Infectious Diseases Society of America (IDSA) updated its treatment guidelines to reflect these newer developments and made therapeutic recommendations based on the known presence of a specific resistance mechanism (21). The M100 33rd edition provides a comment in Table 2A, pointing the laboratory user to consult with ASP when deciding whether to perform phenotypic or genotypic testing for ESBLs to guide therapeutic management. Laboratories may still choose to report ESBLs for infection control or epidemiological purposes but may also report for therapeutic decisions. A similar comment concerning carbapenemase producers was also added to the carbapenem section of M100 Table 2A.

There are several factors that need to be taken into consideration before implementing these new testing and reporting recommendations. One such consideration is whether the laboratory has the workflow and methodology capabilities to assess ESBL and carbapenem resistance mechanisms—either by phenotypic or genotypic methods. False-negative results of phenotypic ESBL testing have been observed due to the coproduction of AmpC beta-lactamases, and false-positive results have occurred among isolates with multiple mechanisms of resistance (22). Phenotypic testing may require additional setup and incubation times and lead to a longer time to results compared to genotypic testing. Certain tests are limited to select species of Enterobacterales, and laboratories using commercial systems may have additional reporting limitations. One must also consider how to report the resistance mechanism accurately and clearly in the laboratory report so that it conveys the correct message to the clinician treating the patient. It is important to note that CLSI M100 does not include specific treatment recommendations. The IDSA treatment guidelines recommend that piperacillin-tazobactam should not be used for ESBL-producing isolates of Enterobacterales, even if the antimicrobial tests are susceptible; however, there is no guidance in the M100 33rd edition that recommends hiding susceptible piperacillin-tazobactam results or changing the interpretation to resistant when an ESBL is detected. These decisions are institution-specific and are to be discussed in collaboration with the laboratory and relevant stakeholders.

The 33rd edition also includes revisions to the general cephems comments (i.e., those in Table 1A footnotes and Table 2A) regarding AmpC beta-lactamase producers. The comments highlight that certain species of Enterobacterales are more likely than others to undergo derepression of their inducible AmpC beta-lactamase within a few days after initiation of therapy with certain third-generation cephalosporins. Derepression is most common with *Citrobacter freundii* complex, *Enterobacter cloacae* complex, and *Klebsiella* (formerly *Enterobacter*) *aerogenes*. Therefore, isolates that initially test susceptible may become resistant. M100 Table 1A lists cefepime as a Tier 1 agent for organisms that are of high risk of derepression of the AmpC beta-lactamase. These revisions align with the current IDSA treatment guidelines for certain genera and species of Enterobacterales that commonly produce AmpC beta-lactamase but may initially test susceptible to ceftriaxone, cefotaxime, ceftazidime, and ceftaroline (21).

### Direct blood culture disk diffusion testing

In 2021, the direct blood culture disk diffusion method was introduced in M100 for organisms belonging to Enterobacterales. The procedure outlined in Table 3E of the M100 33rd edition (now Table 3F of M100 35th edition) entitled “Test for Performing Disk Diffusion Directly from Positive Blood Culture Broth” was first validated for six antimicrobials using standard disk zone diameters when read at 16–18 hours of incubation (23). The method has since been expanded to include early readings (i.e., 8–10 hours of incubation) breakpoints for Enterobacterales for four antimicrobials (i.e., ampicillin, aztreonam, ceftazidime, and ceftriaxone) and new breakpoints for both early and overnight (i.e., 16–18 hours of incubation) readings for ciprofloxacin and meropenem. Additionally, zone diameters for three antimicrobials for *P. aeruginosa* were added: ceftazidime (overnight reading only), ciprofloxacin (early and overnight reading), and meropenem (early and overnight reading). The addition of early reading time points and new breakpoints for *P. aeruginosa* led to the creation of two organism-specific tables: M100 “Zone Diameter Disk Diffusion Breakpoints for Enterobacterales Direct from Blood Culture” (Table 3E-2 in M100 33rd edition) and “Zone Diameter Disk Diffusion Breakpoints for *Pseudomonas aeruginosa* Direct from Blood Culture” (Table 3E-3 in M100 33rd edition). All direct blood culture disk zone diameters are equivalent to the standard disk zone diameters, which are listed in M100 Tables 2A and B, with some exceptions: Enterobacterales and ampicillin early reading; Enterobacterales and ciprofloxacin for both early and overnight readings; Enterobacterales and meropenem for both early and overnight readings; and *P. aeruginosa* and ciprofloxacin early reading. It is critical that laboratories follow the breakpoints as listed in Table 3E of M100 33rd edition (now Table 3F in M100 35th edition) when performing direct blood culture DD testing.

Notably, tobramycin breakpoints were removed from the 33rd edition since the standard DD tobramycin breakpoints had been revised (however, they have since been revised and updated in following M100 editions). When performing direct blood culture DD testing, the organism identification for the Enterobacterales group must be known before releasing an interpretation of the breakpoints, since intrinsic resistance rules must be followed. Specifically, fluoroquinolone breakpoints do not apply to *Salmonella* spp. A past ORWG News Update featured an implementation article on the adoption of this method into the clinical laboratory, including guidance on workflow algorithms. Several additional direct blood culture disk diffusion breakpoints, including *Acinetobacter* breakpoints, were provided in the following versions of M100.

### Alternate test media—Mueller-Hinton Fastidious

CLSI and EUCAST have been working toward harmonizing approaches to AST. As a result, CLSI approved Mueller-Hinton Fastidious (MH-F) agar and broth for *H. influenzae* MIC and disk diffusion testing for select antimicrobial agents. This medium is the standard recommended EUCAST medium for testing fastidious organisms, and an equivalency study was undertaken to assess the performance of this medium as an alternative to *Haemophilus* test medium. MH-F consists of standard Mueller-Hinton (or cation-adjusted MH) with the addition of 5% mechanically defibrinated horse blood and 20 µg/mL nicotinamide adenine dinucleotide. MH-F broth and agar are available through Liofilchem. If used, the test method and breakpoints outlined in the M100 apply to the agents noted in the general comments section of Table 2E. MH-F agar is also an option for disk diffusion testing for *S. pneumoniae*.

### Quality control

Disk diffusion QC range additions in the recent editions of M100 included those for ceftibuten with *E. coli* NCTC 13353 and gentamicin with *N. gonorrhoeae* ATCC 49226. MIC QC ranges were adjusted for imipenem and imipenem-relebactam for *E. coli* ATCC 25922 and *K. pneumoniae* ATCC 700603. QC ranges for TZP were revised for *E. coli* ATCC 25922, as were those for ceftibuten and *E. coli* ATCC 25922, *E. coli* NCTC 13353, *K. pneumoniae* ATCC BAA-1705, and *K. pneumoniae* ATCC BAA-2814. A ceftibuten QC range for *K. pneumoniae* ATCC 700603 was added.

Recommendations for QC of colistin MIC tests were expanded. These included modifications of the QC ranges for *E. coli* NCTC 13486 and *E. coli* ATCC BAA-3170, which are listed in a footnote in Table 5A-1 in M100. Additional recommendations were added to clarify that *P. aeruginosa* ATCC 27853 is intended for routine QC testing, whereas *E. coli* ATCC 25922, *E. coli* NCTC 13486, and *E. coli* ATCC BAA-3170 are supplemental QC strains. A note was added to emphasize that colistin results, including those for QC strains, can be significantly impacted by preparation, handling, and composition of testing materials if methods other than CLSI reference methods described in M07 and M100 are used (24).

There were several new QC ranges added for antimicrobial agents that are not yet approved by the FDA. CLSI will often establish QC ranges early to assist researchers during drug development. Other clarifications include a footnote that was added to explain that *K. pneumoniae* ATCC 700603 may demonstrate two colony morphologies: (i) opaque and cream colored and (ii) translucent, but that either can be used for QC testing. Now that MH-F agar and broth have been approved for testing select agents against *H. influenzae*, recommendations for QC using *H. influenzae* ATCC 49247 with these media were added.

### Quality control test troubleshooting

Several additions were made to the QC troubleshooting guides, which mostly concerned handling and testing of colistin (see above) and avoiding repeated subcultures of QC organisms. Recommendations provided in CLSI M02 or M07 or by AST manufacturers should be followed to ensure optimal performance of QC strains (24, 25). Organism/QC ranges that are most likely to be impacted by inappropriate handling include *P. aeruginosa* ATCC 27853 with carbenicillin and ceftriaxone, as well as *S. pneumoniae* ATCC 49619 and *E. faecalis* ATCC 51299 with all agents. For these, a new subculture from the frozen or freeze-dried stock should be prepared every 2 weeks to prevent loss of strain viability.

## CONCLUSIONS

CLSI continues to refine standards in the field of AST by reviewing data and providing recommendations. Some of the updated or new breakpoints in the CLSI M100 32nd and 33rd editions have not yet been approved by the FDA. The process of breakpoint recognition by the FDA, and the subsequent path to introduce these breakpoints in the various commercial test systems in the United States, can take years. For further reading on the process of breakpoint updates and recent FDA breakpoint updates, we refer readers to two publications by Humphries et al. (26, 27).

A summary of the major changes in the CLSI M100 32nd and 33rd editions and the subsequent actions for each laboratory to consider based on these changes are listed in Table 4. There are many other M100-related educational items besides this minireview produced by the ORWG. A yearly webinar covering changes to the M100 document takes place soon after the publication of the document. A free M100 online self-paced, educational tool is also available and provides in-depth practical guidance on application of this document in the daily workflow in the laboratory; quizzes and questions are peppered throughout to test and cement the user’s knowledge (https://clsi.org/standards/products/microbiology/companion/using-m100/). AST News Updates, released twice a year, include short educational summaries on timely AST topics related to work performed by the AST Subcommittee or the Subcommittee on Antifungal Tests. Archived and current AST News Updates are available on the CLSI website under “Educational Resources” in several languages (https://clsi.org/meetings/susceptibility-testing-subcommittees/). Finally, several key CLSI documents are available free on the CLSI website, including the M100, M27M44S *Performance Standards for Antifungal Susceptibility Testing of Yeasts*, M45 *Methods for Antimicrobial Dilution and Disk Susceptibility Testing of Infrequently Isolated or Fastidious Bacteria*, and M23 *Development of In Vitro Susceptibility Test Methods, Breakpoints, and Quality Control Parameters * (https://clsi.org/meetings/susceptibility-testing-subcommittees/).

**TABLE 4:** Summary of major changes to CLSI M100 32nd and 33rd editions and implementation considerations*^a^*^,^*^b^*^,^*^e^*

M100 32nd and 33rd edition change	Organism/organism group	Implementation considerations^*c*^
Amikacin DD and MIC breakpoints^*b*^	Enterobacterales	Updated breakpoints will improve reporting to reflect current knowledge about dosing
Amikacin DD and MIC breakpoints; now urine only^*b*^	*Pseudomonas aeruginosa*	Application of breakpoints only to isolates obtained from urine will improve reporting to reflect current knowledge about dosing
Amoxicillin-clavulanate DD and MIC breakpoints^*a*^	*Haemophilus* spp.	Updated MIC breakpoints will improve reporting to reflect current knowledge about dosing; insufficient data available to update DD breakpoints, thus DD breakpoints removed
Cefiderocol DD breakpoints ^*a*^	Enterobacterales *Acinetobacter* spp.	Projected test volume; need for testing for isolates encountered and patient population served^*d*^
Cefiderocol DD and MIC breakpoints^*a*^	*Stenotrophomonas maltophilia*	Projected test volume; need for testing for isolates encountered and patient population served^*d*^
Cefiderocol comment added to MIC and DD breakpoints^*b*^	Enterobacterales *Acinetobacter* spp. *P. aeruginosa* *S. maltophilia*	With ASP, determine if comment should be added to reports to explain uncertainties with current cefiderocol tests
Gentamicin DD and MIC breakpoints^*b*^	Enterobacterales	Updated breakpoints will improve reporting to reflect current knowledge about dosing
Gentamicin DD and MIC breakpoint^*b*^	*P. aeruginosa*	Removal of breakpoints reflects current knowledge about dosing
Piperacillin-tazobactam DD and MIC breakpoints; “I” interpretive category now “SDD”^*a*^	Enterobacterales	With ASP, determine if reporting SDD is appropriate for the facility
Piperacillin-tazobactam DD and MIC breakpoints^*b*^	*P. aeruginosa*	Updated breakpoints will improve reporting to reflect current knowledge about dosing
Plazomicin DD and MIC breakpoints^*b*^	Enterobacterales	Limited availability of antimicrobial agent for clinical use and testing^*d*^
Tobramycin DD and MIC breakpoints^*b*^	Enterobacterales *P. aeruginosa*	Updated breakpoints will improve reporting to reflect current knowledge about dosing
Tables 1A–1P: Antimicrobial agents to consider for testing and reporting^*b*^	All	With ASP, determine if antimicrobial agents tested/reported should be modified to include cascade and selective reporting
Reporting comments for ESBLs, AmpCs, and carbapenemases^*b*^	Enterobacterales	With ASP, determine if: (i) additional or new comments should be added to reports; (ii) antimicrobial agents reported should be suppressed; and/or (iii) ESBL or carbapenemase testing/reporting strategies should be modified
Reporting comment for levofloxacin^*b*^	*S. maltophilia*	With ASP, determine if a comment should be added to reports that levofloxacin should not be used alone for antimicrobial therapy
Disk diffusion using positive blood culture broth^*a*,*b*^	Enterobacterales *P. aeruginosa* and select antimicrobial agents	Rapid AST results for GNB from bloodstream infections can potentially impact patient outcomes; reasonable option to consider, particularly if no other rapid AST/AR detection method used for blood cultures positive with GNB
MH-F as alternative media for DD and MIC testing^*b*^	*Haemophilus influenzae* and select antimicrobial agents	Optional media for testing
QC strain troubleshooting^*b*^	*Klebsiella pneumoniae* ATCC 700603	ATCC indicates isolate may demonstrate two colony morphologies on subculture and either can be used for QC testing
QC strain troubleshooting^*b*^	*P. aeruginosa* ATCC 27853 *Streptococcus pneumoniae* ATCC 49619 *Enterococcus faecalis* ATCC 51299	Susceptibility characteristics for these QC strains are not stable; new subculture from stocks should be prepared every 2 weeks
Colistin QC troubleshooting^*b*^	All	CLSI recommendations for handling colistin should be followed to avoid erroneous results
Ceftibuten DD and MIC QC ranges^*b*^	*Escherichia coli* ATCC 25922 *E. coli* NCTC 13353 *K. pneumoniae* ATCC BAA-1705 *K. pneumoniae* ATCC BAA-2814 *K. pneumoniae* ATCC 700603	New data support updated or additional QC ranges
Colistin MIC QC ranges^*a*^	*E. coli* NCTC 13486 *E. coli* ATCC BAA-3170	New data support updated QC ranges
Gentamicin QC range^*b*^	*Neisseria gonorrhoeae* ATCC 49226	Additional QC range
Imipenem MIC QC ranges^*a*^	*E. coli* ATCC 25922 *K. pneumoniae* ATCC 700603	New data support updated QC ranges
Imipenem-relebactam MIC QC ranges^*a*^	*E. coli* ATCC 25922 *K. pneumoniae* ATCC 700603	New data support updated QC ranges
Piperacillin-tazobactam MIC QC range^*b*^	*E. coli* ATCC 25922	New data support updated QC ranges

^*a*^ Change occurred in M100 32nd edition.

^*b*^ Change occurred in M100 33rd edition.

^*c*^ Prioritization is based on the authors’ opinion and should be discussed at the institutional level with physicians, pharmacy, antimicrobial stewardship teams, and hospital leadership. Refer to the text for details and references.

^*d*^ Based on the projected test volume, consider submission to an outside laboratory, such as a reference or public health laboratory, when results are needed.

^*e*^ AR, antimicrobial resistance; ASP, antimicrobial stewardship program; DD, disk diffusion; GNB, gram-negative bacilli; I, intermediate; MH-F, Mueller-Hinton Fastidious; ESBL, extended-spectrum beta-lactamase; and S, susceptible.
